# Structural Details of BaTiO_3_ Nano-Powders Deduced from the Anisotropic XRD Peak Broadening

**DOI:** 10.3390/nano11051121

**Published:** 2021-04-26

**Authors:** Iuliana Pasuk, Florentina Neațu, Ștefan Neațu, Mihaela Florea, Cosmin M. Istrate, Ioana Pintilie, Lucian Pintilie

**Affiliations:** National Institute of Materials Physics, 405A Atomistilor Street, 077125 Magurele, Romania; iuliana.pasuk@infim.ro (I.P.); florentina.neatu@infim.ro (F.N.); stefan.neatu@infim.ro (Ș.N.); mihaela.florea@infim.ro (M.F.); cosmin.istrate@infim.ro (C.M.I.); ioana@infim.ro (I.P.)

**Keywords:** nano-BaTiO_3_, size and strain anisotropy, surface relaxation, tetragonal deformation, temperature-dependent XRD and Raman measurements

## Abstract

In this study, nano-BaTiO_3_ (BTO) powders were obtained via the solvothermal method at different reaction times and were investigated using transmission electron microscopy (TEM), X-ray diffraction (XRD) and Raman spectroscopy. The results were compared with those obtained for a larger crystallite size BTO powder (BTO-m). The sizes of the cuboid crystallites (as determined by XRD and TEM) ranged from about 18 to 24 nm, depending on the reaction time. The evolution with temperature of the structure parameters of nano-BTO was monitored by means of X-ray diffraction and Raman spectroscopy and no signs of phase transition were found up to 170 °C. Careful monitoring of the dependence of the XRD peak widths on the *hkl* indices showed that the effect of the cubic crystallite shape upon the XRD peak widths was buried by the effect of hidden tetragonal line splits and by anisotropic microstrain. The good correlation of the line widths with the tetragonal split amplitudes, observed especially for BTO-m above the transition temperature, indicates tetragonal deformations, as also revealed by Raman spectroscopy. The large anisotropic microstrain shown by the nano-powders, which had a maximum value in the <100> directions, was considered evidence of the phenomenon of surface relaxation of cubic crystallites edged by {100} faces. The observed behavior of the nano-BTO structures with increasing temperature may suggest a correlation between the surface relaxation and tetragonal deformation in the nano-cubes. The experimental results for both nano-BTO and mezoscale-BTO are in agreement with the core-shell model.

## 1. Introduction

The latest results have demonstrated that nanocrystals represent interesting candidates for developing materials with high electrical properties due to their crystallinity, size and shapes, which are therefore of key general importance [1].

BaTiO_3_ (BTO) is a well-known and intensively studied ferroelectric material in various forms—single crystals, bulk ceramics, thin films and nanocrystals. Its ferroelectric, dielectric, piezoelectric and pyroelectric properties make it attractive for a variety of applications, ranging from non-volatile memories to tunable microwave devices and super-capacitors for energy storage.

BTO nanocrystals of cuboid shape can be prepared by relatively low-cost chemical methods. The cuboid shape is the most desirable morphology due to its ability to form a close-packed structure, in this way delivering a higher number of contacted nanoparticles than other morphologies. Furthermore, the interest in BTO nanocubes was triggered by the fact that these nanostructures can be assembled in ordered thin films, which can be used in applications in field effect transistors (as gate oxides) or in multilayer capacitor structures due to their large dielectric constants and low dielectric losses. The existing literature suggests that the properties of the films can be tuned by adjusting the size of the component BTO nanocubes. According to the nanosize effect, a decrease in BTO crystallite size induces a decrease in their dielectric constant [2,3]; nevertheless, there are studies which demonstrate that BTO nanocrystals of cubic shapes smaller than 15 nm maintained their ferroelectricity [4,5].

Studies have been performed on nanocubes of different sizes, usually around 20 nm or above. Considering the high potential for application of the films derived from BTO nanocubes, it is important to study their structural properties at lower sizes and at different temperatures and to establish if ferroelectricity is still present or not.

In this respect, the choice of the preparation method is very important, as it can play an important role on the structural, textural and electrical properties of the final material. Therefore, the 3D assemblies of BTO crystallites must be highly ordered to maintain the initial properties of the nanocrystals. Moreover, for this process a narrow shape and size distribution is needed and usually the self-assembly technique is preferred in order to obtain large assemblies [6].

BTO can be prepared using different methods [7]. Thus, a large variety of BTO nanostructures, such as nanodots [8,9], nanoparticles [10], nanobowls [11], nanorods [12], nanocubes and nanowires [13], possessing different crystalline phases (e.g., cubic [14,15], tetragonal [16] and polycrystalline [17]), have been produced through various chemical approaches, such as organometallic [18,19], solvothermal/hydrothermal, templating [14,15,17,20], molten salt [14,21] and sol-gel methods [22,23].

The structure of BaTiO_3_ is very complex, despite the apparent simplicity of being based on cubic perovskite, and it has been intensively studied both experimentally and theoretically. Many studies have focused on BTO nano-powders to determine whether the structure is cubic or tetragonal with small tetragonality, mainly aiming to clarify the occurrence of spontaneous polarization in nano-powders of BTO [24,25,26]. The effects of size upon the ferroelectric and dielectric properties of BTO are attributed to grain-boundary effects. To explain these observations, the core-shell model has been proposed. According to this model, for large crystallite monodomain BTO, the tetragonal core structure has a cubic surface structure [27,28]. Conversely, for nano-size BTO, the core structure is cubic, and the surface structure is tetragonally deformed. Since the thickness of the shell is independent of the size of the core, the model is similar to that of surface relaxation [29,30].

The ferroelectricity decreases with a decrease in the particle size and disappears below the critical size. This phenomenon has been explained by the increase in the volume fraction of the surface cubic layer with decreasing particle size [28].

Here, we present the structural properties of two nano-BTO powders with crystallite sizes of 18 nm and 24 nm, prepared by means of solvothermal chemical synthesis at different reaction times. A commercial BaTiO_3_ powder, tetragonal at room temperature, has also been studied to highlight the effects of size upon the structural characteristics. The study was based on X-ray diffraction (XRD), Raman spectroscopy and transmission electron microscopy (TEM) investigations. The XRD and Raman analyses were performed at temperatures ranging from room temperature up to the ferro-paraelectric phase transition of the bulk BaTiO_3_, which is between 120 °C and about 130 °C [31,32]. The crystallite sizes, lattice constants and microstrain, and their evolution with temperature, were determined through Pawley fitting of the diffractograms, approximating the nano-BTO structures with cubic perovskites with isotropic size and microstrain. After that, interesting results concerning the size (shape) and microstrain anisotropy and tetragonal deformation were obtained by carefully monitoring the peak widths’ dependence on the Miller indices. The microstrain anisotropy is discussed as evidence of the surface relaxation of the cube shaped BTO nanocrystals. This study revealed non-obvious structural features of both nano-BTO and large crystallite BTO through simple and easily accessible methods. The thermal expansion coefficients are determined based on the temperature dependence of the lattice constants, a quantity that could be important for power devices dissipating large amounts of heat.

## 2. Materials and Methods

### 2.1. Materials

The following reagents were purchased from Merck (Merck KGaA, Gernsheim, Germany) and used without any further purification: barium acetate (99.999% trace metals basis) Chemical Standard Service (CAS) no. 543-80-6, sodium hydroxide (reagent grade, ≥98% anhydrous) CAS no. 1310-73-2, titanium (IV) isopropoxide (99.999% trace metals basis) CAS no. 546-68-9, 2-propanol (analytical standard, anhydrous) CAS no. 67-63-0, oleic acid (≥99%) CAS no. 112-80-1. To overcome any contamination of our BTO samples, only deionized water (resistivity of 18.2 MΩ cm measured at 25 °C) was used in all preparation steps.

### 2.2. BTO Preparation Procedure

For the preparation of BTO nanocrystals, a solvothermal method reported elsewhere [20] was employed. This is an easy and cost-efficient technique, known to lead to the formation of different organized structures that are difficult to obtain using other existing procedures. Since the formation of BTO by means of the hydrothermal method can be influenced by the properties of the Ti-precursors and because the solvent also plays an important role in the nucleation process, we slightly modified the original method. For instance, we used a different Ti precursor, Ti isopropoxide, instead of Ti(OBu)_4,_ and 2-propanol instead of *n*-butanol.

Thus, to obtain approx. 1 g of BTO sample, four solutions were initially prepared: (a) solution 1, which contained 0.5 g Ba(CH_3_COO)_2_ dissolved in 10 mL H_2_O (0.2 M); (b) solution 2, which contained 1 g NaOH added into 10 mL H_2_O (2.5 M); (c) solution 3, containing 0.5650 g Ti isopropoxide (TIPO) solubilized in 10 mL 2-propanol anhydrous (0.2 M); and finally (d) solution 4, which contained 5 mL of oleic acid dissolved in 10 mL of 2-propanol anhydrous (1.5 M). First, solutions 1 and 2 were mixed to reach a mixture with a pH of 14 with the formation of a white precipitate. Later, the third solution containing the Ti precursor was dropwise added to the obtained mixture and the pH dropped to 4. Lastly, the fourth solution, containing the oleic acid, was added to the aforementioned mixture and finally a white creamy precipitate was formed. The suspension was stirred vigorously for another 30 min and then transferred into a stainless-steel autoclave provided with a Teflon liner. The autoclave was treated at 180 °C for different time intervals. Two reaction times were studied: 3 h and 18 h, respectively. Thus, the resulting BTO powders are referred to below as BTO-3h and BTO-18h, respectively, whereas the commercial BTO is referred to as BTO-m. At the end of the reaction the precipitate was separated via centrifugation and carefully washed several times with ethanol and then dried in air at 100 °C overnight.

Due to the fact that traces of BaCO_3_ were identified in the X-ray diffraction patterns and Raman spectra, additional washing steps (at least 4) with 5% *v*/*v* acetic acid were performed. After this step, the samples were dried again overnight at 100 °C in air.

### 2.3. Characterization of the BTO Samples

The crystal structure, vibrational properties and morphology of the BTO powders were studied using XRD, Raman spectroscopy and TEM.

The XRD measurements were performed using a Rigaku-SmartLab X-ray diffractometer (Rigaku Corporation, Tokyo, Japan) with a copper anode X-ray tube powered at 40 kV and 40 mA, in Bragg–Brentano geometry, using a Hypix detector in 1D mode. The powders were measured in zero background silicon sample holders with a cavity height of 0.1 mm. The initial powders were scanned in the angular range 2θ = 15–90°. Temperature-dependent XRD measurements were performed by using a DHS 1100 temperature chamber (Anton Paar GmbH, Graz, Austria), in normal atmosphere, from room temperature up to 140 °C, with a temperature step of 10 °C (5° for BTO-m around the transition temperature) and a stabilization time of 5 min before starting the measurement at each temperature. The temperature-dependent XRD measurements were performed in the 2θ range 15–80° for BTO-m, and 28–47.5° for BTO-3 h and BTO-18h, with scan speed = 0.5°/min and step size = 0.01°. The integral line breadths were determined using EVA v.13.0 software (Bruker AXS Inc., Madison, WI, USA) after removing the contribution of the Cu K_α2_ spectral line of the X-ray tube using the same software. The whole powder pattern fitting was performed using the Bruker-TOPAS v.3 program (Bruker AXS Inc., Madison, WI, USA) in the fundamental parameters approach. During the refinement, the sample displacement parameter was refined for each diffractogram, and the goniometer offset was fixed at zero.

The Raman measurements were performed using a LabRAM HR Evolution confocal Raman microscope (Horiba Jobin Yvon, Kyoto, Japan) with an experimental configuration consisting of a 100× objective lens and using a 633 nm He-Ne laser. The temperature-dependent spectra were collected in the range of 23–170 °C, with a heating rate of 50 °C/min, using a THMS600 heating microscope-stage (Linkam Scientific Instruments, London, UK). Spectra were obtained at 1 cm^−1^ resolution using an 1800 lines/mm grating, aligned to the wavenumber region between 200–800 cm^−1^ and averaging 5 multiple accumulations of 20 s integration time per spectrum using an air-cooled Syncerity OE charged coupled device (CCD) detector. Data analysis of raw spectra was subsequently performed using Horiba Jobin Yvon’s LabSpec6 software. Baselines were subtracted from all of the spectra presented, and individual spectra were normalized to the highest intensity of 1.

The TEM images were obtained using a JEM ARM 200F (Jeol Ltd., Tokyo, Japan) analytical transmission electron microscope, at an acceleration voltage of 200 kV. The samples for TEM analysis were prepared by dripping on a microscopy grid, deposited initially on a carbon membrane, a few drops from the BTO suspension obtained by immersion of the powder of BTO in cyclohexane.

## 3. Results and Discussion

### 3.1. TEM Investigations

**Figure 1:**
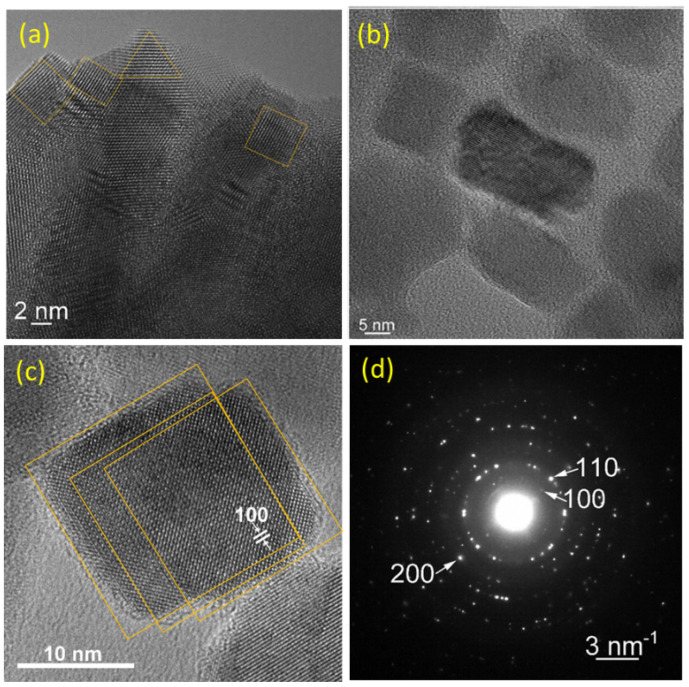
Electron microscopy (TEM) images of the nanocubes. High-magnification view of non-dispersed (**a**) and completely dispersed particles of, nano-BaTiO_3_-18h (BTO-18h), (**b**) partially dispersed BTO-3h (**c**) and the corresponding SAED diagram (**d**). The faint lines are drawn to guide the eye.

High-magnification TEM images of the prepared nano-BTO powders are presented in Figure 1 at different dispersion stages, since sample preparation is crucial in TEM analysis. For instance, when powders were not completely dispersed in cyclohexane (see Figure 1a BTO-18h sample), one can distinguish particles with smooth sides, which join at 90°. In addition to cubic or rectangular shapes, triangular shapes can also be observed. The triangular shapes are probably truncated cubes, with corners cut with {111} planes which can become at the limit octahedrons, bordered only by {111} faces. The elongated particles are presumable edge-cut cubes, with the planes {110} up. Unfortunately, these images do not allow size measurements because of shadows caused by the overlapping grains. When the particles are well dispersed in cyclohexane, the faces are no longer smooth, and no sharp edges and corners can be seen (see Figure 1b). This suggests that the crystallites are damaged during the dispersion process. This can be well seen in Figure 1c, showing almost completely dispersed particles for the BTO-3h sample. This image allowed us to observe that the interplanar spacing parallel to the cube sides corresponds to the {100} planes of the cubic BTO structure. The SAED analysis also confirmed the cubic structure of the BTO crystallites (see Figure 1d). The particle lengths found by TEM for both samples were about 15–20 nm.

### 3.2. XRD Analysis

BaTiO_3_ is commonly tetragonal at room temperature, from about 0 °C to about 120 °C, cubic above this limit, and orthorhombic below, down to −90 °C; below −90 °C it is rhombohedral [31,32,33].

**Figure 2:**
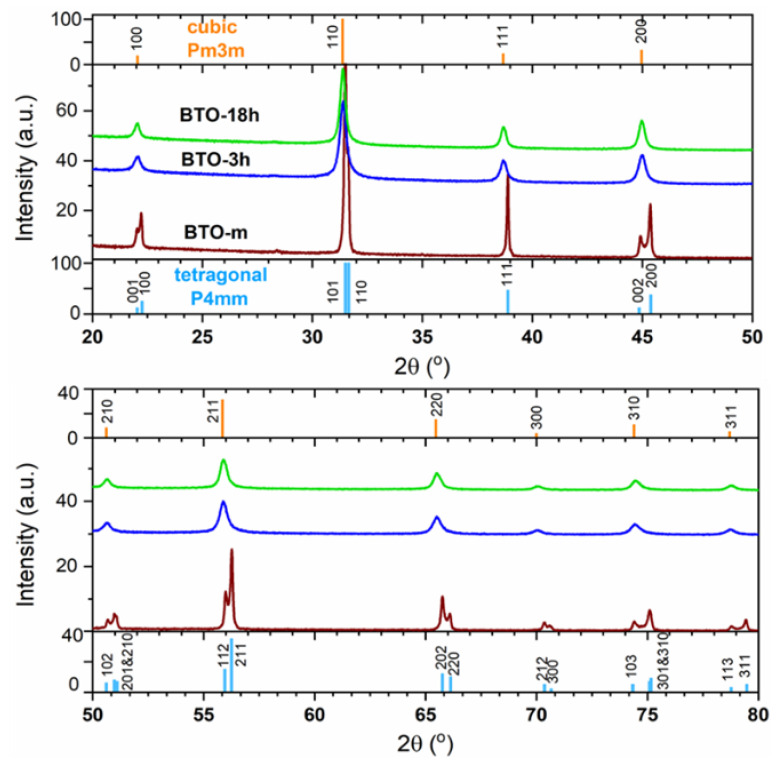
The X-ray diffraction (XRD) patterns of the BTO samples and the line indexing with tetragonal and cubic BTO. The sticks indicate cubic BaTiO_3_ according to ICDD-PDF#01-084-9618 (a = 4.029 Å) and tetragonal BaTiO_3_ according to ICDD-PDF# 00-005-0626 (a = 3.994 Å, c = 4.038 Å). The K_α2_ contribution was removed using the software. The intensity of BTO-m was reduced 1000 times to scale with the nano-BTO patterns. The patterns are vertically shifted.

The XRD phase identification showed that both prepared nano-BTO samples matched the cubic perovskite structure, space group Pm3m, whereas BTO-m is tetragonal, space group P4mm (see Figure 2). The diffractograms are represented in two panels for the visualization of the tetragonal line splits. The intensity scales are lower at the high-angle panel to allow the visualization of the small-intensity peaks at higher angles.

However, in the case of nano-BTO the identification is uncertain, since, due to the large line widths caused by the small crystallite sizes, the characteristic tetragonal line splittings may not be noticeable. Many studies have revealed that the structure of nano-BTO is far from a perfect cubic perovskite [25,26,28].

#### 3.2.1. Isotropic Approximation

##### The Structure at Room Temperature

**Figure 3:**
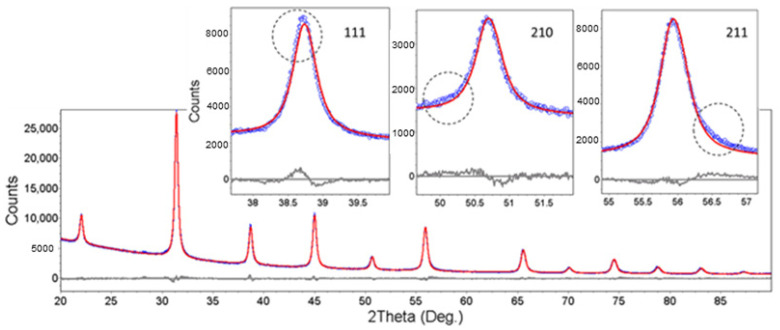
The XRD pattern of BTO-3h fitted using the Pawley method; blue—experimental points, red—calculated curve, gray—difference curve.

To determine the average crystallite sizes and the lattice constants, we assumed in the first approximation that the structure of the nano-BTO was that of a perfect cubic perovskite. A fairly good match of the XRD patterns was obtained by applying the Pawley refinement approach [34] in Bruker-TOPAS software, assuming a cubic Pm3m structure. This method does not involve a structure model containing the atomic positions and site occupancies in the unit cell. It only fits the line profiles, providing exact peak positions and widths, based on the space group and on a model of line width dependence on line positions. The diffractograms were well matched; however, some non-negligible differences between the calculated and experimental line profiles were noticed and are highlighted in the insets of Figure 3. The deviations of the experimental peak profiles from the simulated ones may have many origins, of which we especially suspected the pronounced crystallite shape anisotropy, with the possibility of the co-existence of different external shapes (cubes, parallelepipeds, octahedra) in different proportions, with possible size dispersions. Moreover, the lattice symmetry may differ from the cubic one, or there could be a mixture of different BTO polymorphs. Trying to take into account all the possible effects, the analysis could become very complex [35].

**Figure 4:**
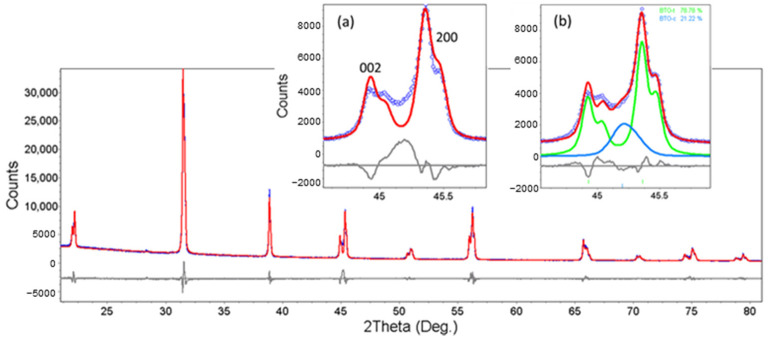
The XRD pattern of BTO-m fitted using the Pawley method with a tetragonal phase (main panel and inset (**a**)), and fitted using the Rietveld method with tetragonal and cubic phase (inset (**b**)). Blue—experimental points, red—calculated curve, gray—difference curve, green—simulated pattern of tetragonal BTO, light blue—simulated pattern of cubic BTO.

For BTO-m, the fit is much worse, especially due to the increased intensity between the close tetragonal lines (Figure 4, inset a). As we intended to include BTO-m in the study only as a reference mainly for the lattice parameters, the fitting focused on matching the tetragonal lines’ positions as accurately as possible. This type of fitting was the basis of the results presented in the tables and graphs below. However, according to a simplified core-shell model (without the “gradient lattice strain layer” proposed by Hoshina et al. [28]), the tetragonal structure is surrounded by a shell with a cubic structure. By adding a cubic BTO phase, the matching becomes better (Figure 4, inset b). The concentration of the cubic phase is estimated at 21–22% by quantitative Rietveld analysis, and the cubic lattice parameter is a = 4.009 Å.

**Figure 5:**
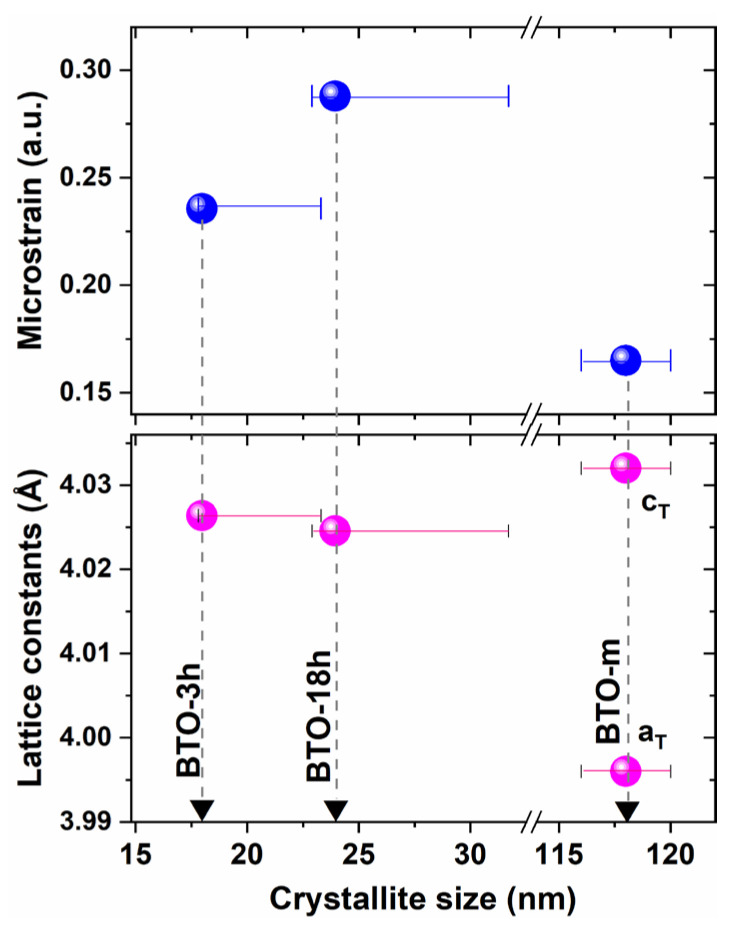
The structure parameters of the samples obtained by means of XRD, assuming perfect cubic perovskite structures for the nano-BTO samples and a single-phase tetragonal structure for BTO-m.

The results obtained by means of Pawley whole pattern fitting are presented in Table 1, and are represented in Figure 5. One has to recall that size and microstrain broadening usually occur together, especially for small crystallites. Microstrain is usually expressed as <ε^2^>^1/2^, where ε = Δ*d*/*d* stands for the dispersion of the relative variation of the interplanar spacings, and the angle brackets stand for ‘average’. If the contribution of the microstrain is neglected, the coherence domain sizes are systematically underestimated. These minimum values of the crystallite sizes are also given in Table 1. The maximum values included are the cube sizes, determined taking into account the anisotropy of the microstrain, according to the analysis presented in Section 3.2.2 of the XRD results.

Summarizing the results, the prepared nano-BTO samples were constituted of crystallites with average sizes of about 18 nm for BTO-3h and 24 nm for BTO-18h, as a function of the reaction time, and both can be considered as having cubic lattices with large microstrain. The microstrain may originate, for example, from the different structure of the crystallite boundary or inclusions in the lattice [36]. The commercial BTO powder, declared to be micrometric (BTO-m), has a tetragonal structure, therefore it is in a ferroelectric state. For ferroelectrics, the coherence length is determined by the ferroelectric domain sizes. The local lattice distortion, expressed by the quantity “microstrain”, was found to be quite large for the tetragonal phase of this powder (for well crystallized powders, applying the same software and fitting procedure, this parameter is commonly found to be at least 10 times smaller). This relationship might be expected, given that this single tetragonal structure also approximates the gradient lattice strain supposed by the surface relaxation or the core-shell model, which are discussed below.

##### The Structure above Room Temperature

**Figure 6:**
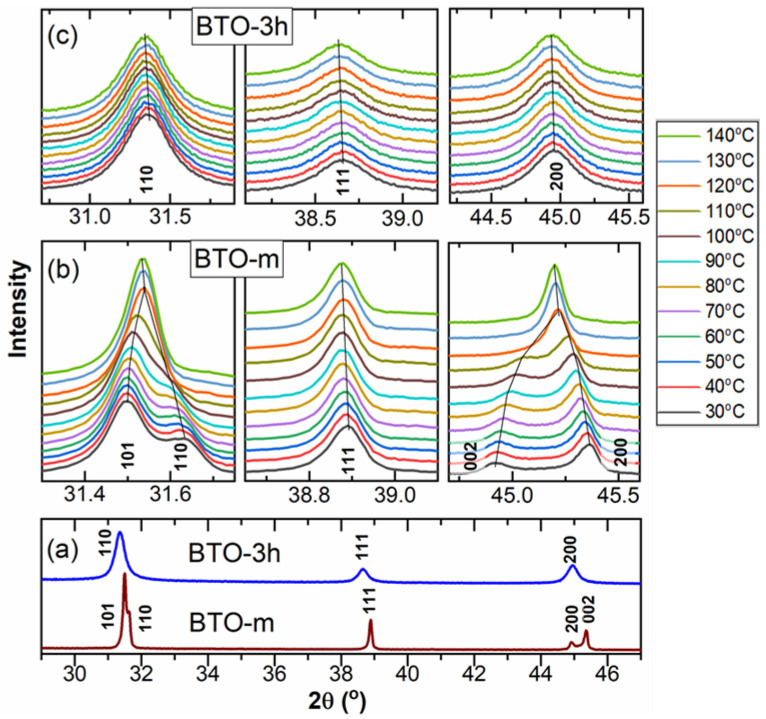
The XRD patterns of BTO-m and BTO-3h obtained at different temperatures. (**a**) The whole 2θ range at 30 °C; zoomed views of the peaks at different temperatures for BTO-m (**b**) and BTO-3h (**c**).

XRD measurements at temperatures from room temperature up to 140 °C for the nano-powders (exemplified by BTO-3h) and for BTO-m are presented in Figure 6. At room temperature, the diffractograms look very different (Figure 6a) but become similar at 140 °C. In the case of the BTO-m sample, the tetragonal splitting decreases with temperature, until the two lines merge, marking the phase transition, at approximately 120 °C (Figure 6b). The peaks of the nano-BTO samples do not show noticeable changes with temperature, except for a slight shift to small angles caused by the thermal expansion (Figure 6c).

**Figure 7:**
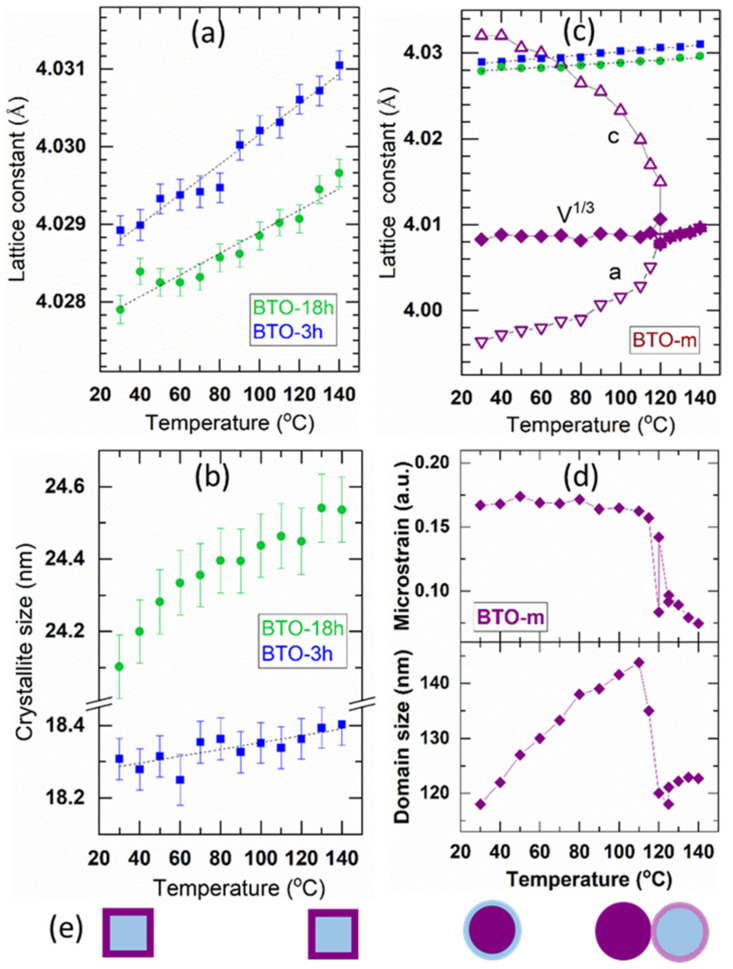
The structure parameters of the analyzed samples as a function of temperature; the cubic lattice constant (**a**) and the average crystallite size (**b**) of the prepared nano-BTO powders; the lattice constants (**c**) and the coherence domain size and microstrain (**d**) of BTO-m; (**e**) sketches of the crystallites at the corresponding temperatures; blue—cubic phase, purple—tetragonal phase.

The XRD data were analyzed as above by means of whole powder pattern fitting using the Pawley approach in order to refine the structure parameters. For the prepared nano-BTO samples the microstrain was kept fixed at the value obtained for the sample at room temperature, considering that the narrow 2*θ* range measured with temperature does not allow the adequate separation of size and microstrain effects. The resulting values are represented as a function of temperature in Figure 7.

The tetragonal-cubic phase transition is well marked for the BTO-m powder at about 120 °C, at which the *a* and *c* lattice constants of the tetragonal phase abruptly became equal (Figure 7c). The V^1/3^ plot presented in this graph is for the lattice constant of a cubic lattice having the same unit cell volume as that of the tetragonal lattice, $V=a_{T}^{2}\;c_{T}$. The lattice parameters of the prepared samples, BTO-3h and BTO-18h, determined assuming a perfect cubic structure, vary linearly with temperature without showing any change in the slope up to 140 °C (Figure 7a). The lattice constant of the pseudo-cubic nano-BTO powders is considerably larger than V^1/3^ for BTO-m, which is in agreement with the literature [13,36], and it is larger for the sample of smaller crystallite size, BTO-3h, which is in agreement with the surface relaxation model for the nano-sized BTO structures [29]. The lattice constant of the nano-BTO samples increases practically linearly with temperature (Figure 7a). The graphs were superimposed over the corresponding graph of BTO-m to facilitate the comparison of sizes and slopes. The slope is directly related to the linear thermal expansion coefficients and allows its determination. The values obtained are presented in Table 2. The thermal expansion coefficients are greater for nano-BTO than for BTO-m and decrease as the nanocrystallite size increases.

Figure 7b shows that the increase in crystallite size is insignificant for the nano-BTO samples (at most 4 Å, of which almost 10% is thermal expansion, and the rest is probably due to the ordering of the atoms at the boundary). In the case of BTO-m, the situation is completely different (see Figure 7d). The coherence domains (which are the ferroelectric domains in the case of ferroelectrics) increase up to the transition temperature by about 20 nm and, shortly before the phase transition, decrease suddenly to approximately their initial size. The lattice strain also decreases sharply close to the transition temperature. Assuming that the crystallites are single ferroelectric domains (in the case of BTO, 120 nm is about the upper limit of the crystallite size that develops a single ferroelectric domain [37]), we propose the following scenario to explain this behavior: As the temperature increases, and the tetragonality of the tetragonal core structure reduces, it gradually embeds the surrounding cubic shell (this is supported by the fact that in the cubic shell, there is probably a gradient of tetragonality decreasing towards the surface), until the core and the whole shell become a single coherence domain, with a structure very close to the cubic one. In the core-shell model for BTO, the shell thickness has been estimated at different values, from 5 nm [27] to about 10–15 nm [28], the thickness being independent of the size of the crystallites. In our case, the increase in the coherence length by about 20 nm indicates a thickness of 10 nm at each opposite side, a value that falls within the range of the shell thicknesses reported in these references. Therefore, the increase in the coherence length with temperature could be explained in the way presented above. The transition begins due to the occurrence of cubic-BTO nuclei of the size of a couple of unit cells near the crystal surface [38]. From the point of view of XRD, this leads to the destruction of the coherence of the surface structure, and therefore to the decrease in the size of the coherence domain. Upon heating, the transition occurs in the whole volume. Given that the coherence domain decreases, we can speculate that the shell’s fragmented structure transforms more slowly. This outer structure may contain the nanometer size domains revealed for the first time by TEM studies in 1984 by Bursill and Lin. [39]. This intermediate form between the tetragonal and cubic structure of BTO has a sharp onset temperature and occurs over a range of temperatures, being quite stable up to 140 °C. A short-range order structure occurring around the phase transition, which deviates from the average structure of BTO, has also been demonstrated by X-ray topography [40] and XRD pair distribution function analysis [26]. The evolution of the crystallite size suggested by our experimental results is sketched in Figure 7e. This tentative interpretation needs further confirmation by dedicated studies.

#### 3.2.2. The *hkl*-Dependent XRD Peak Broadening: Evidence of Tetragonal Deformation, Cubic Shape and Surface Relaxation

All the results presented so far were based on the assumption of a cubic perovskite structure with isotropic crystallite shapes for nano-BTO. However, we obtained clear evidence of the tetragonal phase in the prepared nano-BTO samples via Raman spectroscopy, as we demonstrate below. On the other hand, the literature extensively deals with the tetragonal deformation and deviations from the ideal perovskite structure of nano-sized BTO [25,26].

The small deviations, shown in Figure 3, of the calculated peak profiles from the experimental ones, indicate that the real structure differs from the assumed model. We propose the following major reasons for the observed deviations: the crystal lattice symmetry, which is different from the cubic one, and the microstructure anisotropy, i.e., crystallographic direction-dependent crystallite size and microstrain.

In this study we tried to unveil structural details by studying the dependence of the peak widths on the Miller indices. We chose for this the integral breadth (area of the peak/peak height) instead of the full width at half-maximum, supposing that this is less affected by the profile details determined by the overlapping of several effects.

The starting hypotheses were: (1) The symmetry of the crystal lattice may be tetragonal; therefore, even if the peaks are not split, the peak profiles could be modified due to the overlapping of several lines of different intensities. (2) The crystallite shape is predominant cubic, faced by {100} planes, or derived from cubic, such as an octahedron shape, as suggested by the TEM images shown in Figure 1; this shape anisotropy causes anisotropic XRD line broadening, accentuated by the small crystallite sizes. (3) In the large-crystallite-size BTO, BTO-m, the crystallite shapes are no longer regular, and there is no anisotropic line broadening due to the size effect; therefore, we intended to use BTO-m recorded at 140 °C (above the phase transition) as a reference for the line broadening produced by an isotropic cubic phase BTO, and thus with peak widths varying monotonously with the line positions.

**Figure 8:**
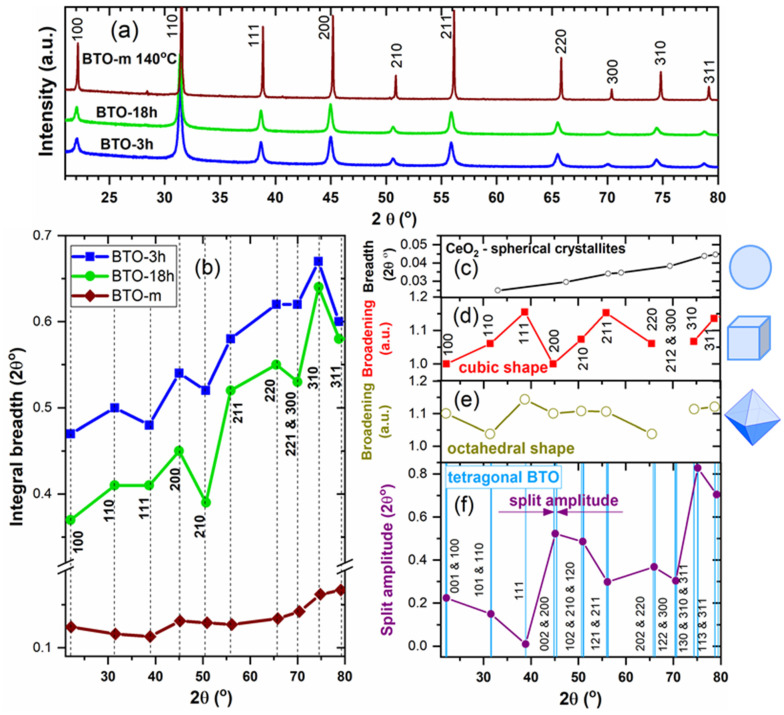
(**a**) The XRD patterns of the nano-powders compared with BTO-m in cubic phase at 140 °C (the K_α2_ contribution has been removed and the patterns are shifted vertically); (**b**) the integral widths of the nano-powders and of BTO-m at 140 °C as a function of Miller indices; (**c**) line broadening due to isotropic microstructure; (**d**,**e**) theoretical line broadening caused by cubic and octahedral external shapes, respectively, according to Table 3; (**f**) tetragonal split amplitude.

The XRD pattern of BTO-m recorded at 140 °C is presented comparatively to the patterns of nano-BTO powders at room temperature in Figure 8a, and the peak widths as a function of line positions and of the Miller indices are represented in Figure 8b. It can be observed that the widths are strongly *hkl*-dependent and it is remarkable that the variation follows the same pattern for both nano-BTO samples. Unexpectedly, one can also notice an *hkl* dependence for BTO-m at 140 °C.

In an attempt to explain the variation of the peak width as a function of *hkl*, we compared the experimental plots from Figure 8b with several reference graphs of the line width as a function of line position, as shown in Figure 8c–f. We considered the following effects:

(1) A monotonous increase in the line widths caused by an isotropic microstructure and instrumental broadening [41]. The plot was obtained based on the diffractogram of a heat-treated ceria powder (Figure 8c). The general increasing trend in the peak widths visible in Figure 8b is due to this component.

(2) The effect of the cubic or octahedron shape of the crystallites. According to the Scherrer equation, the width of a diffraction line with indices *hkl* is inversely proportional to the average column length perpendicular to the (*hkl*) lattice planes (volume weighted crystallite sizes). These were calculated by Wilson [42] for a crystal with a cubic lattice having several external shapes: a cube bounded by {100} faces, a tetrahedron and an octahedron bounded by {111} faces, measured perpendicular to different crystallographic planes. Later, these calculations were extended to nanocrystals with any polyhedral shape, allowing the calculation of the corresponding powder diffraction pattern [43]. We consider here only cubic and octahedral shapes, the latter because octahedra seem to be revealed in the TEM images (Figure 1a). Wilson expressed the calculated values as fractions (cube side)/(average column length perpendicular to the (*hkl*) planes) for cubes, and (octahedron edge-length)/(average column length perpendicular to the (*hkl*) planes) for octahedrons. These values are listed in Table 3. Due to the inverse proportionality between the line width and the corresponding crystallite size, the above fractions are proportional to the *hkl* line widths. The values are plotted in Figure 8d for the cubic and in Figure 8e for the octahedral external shape. (These curves do not exhibit the monotonous increase in width due to the instrument.) In a cubic lattice, the planes (300) and (212) have the same interplanar distance, having the same value of the expression *h*^2^ + *k*^2^ + *l*^2^, hence the diffraction lines are perfectly superimposed. Therefore, these points were not taken into account in the Figure 8d,e. Comparing the experimental plots in Figure 8b with these theoretical broadenings determined by the external shape, no correlations were observed. For example, line 111 should be the widest due to the size effect in both cubic and octahedral case, but it was found, on the contrary, to be the narrowest for our nano-BTO samples. Hence, one could conclude that the main source of the observed variations in the peak widths is not the crystallite shape, or the crystallites are not cubic in any of the nano-powders.

(3) Expected peak broadenings due to hidden tetragonal splits. We tried to quantify the effect of possible hidden tetragonal peak splits upon the peak widths by calculating the 2θ distance between two or more close tetragonal lines of the reference tetragonal BTO phase (the same reference of which the lines are represented in Figure 2; the tetragonality is 1.011). We named these values ‘tetragonal split amplitude’ and represented them as a function of the line positions in Figure 8f. The 111 line is the only simple line (not split) in the case of tetragonal BTO, and therefore it should be the narrowest if the tetragonal contribution is important. One can observe that for both nano-BTO samples the width of this line is a local minimum, whereas the next peak, 200, which has a large tetragonal split amplitude, is broader. This could be a sign of tetragonal deformation.

**Figure 9:**
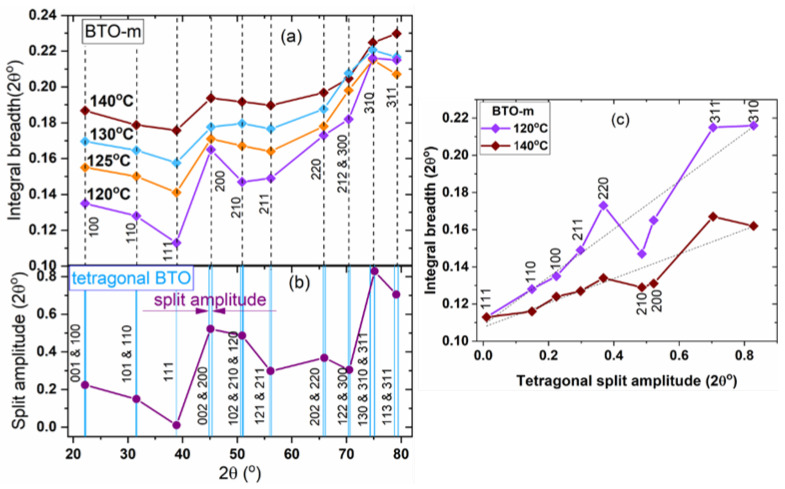
Line widths of BTO-m above the phase transition temperature (**a**); compared with the tetragonal split amplitude (**b**); the linear correlation between the peak widths and the tetragonal split amplitude (**c**).

For the nano-BTO samples, no clear conclusions can be drawn based on the graphs in Figure 8 regarding the origin of the anisotropic peak broadening, because many effects are superimposed. Instead, in the case of BTO-m above the transition temperature there is an undoubted correlation between the experimental peak widths and the split amplitude, as can be seen in the zoomed view presented in Figure 9a. There is a linear dependence between the peak widths and split amplitude, with only a few points significantly deviated from the straight line (Figure 9c). This is clear evidence of the presence of tetragonal distortion above the transition temperature. With increasing temperature, the plots become smoother, indicating that the tetragonal distortion diminishes. This is in agreement with the Raman spectra presented below, which show that for BTO-m, the characteristic lines of the tetragonal phase severely decrease at the tetragonal-cubic transition and gradually decrease in intensity as temperature increases above the transition. The tetragonal imprint above the transition temperature may be due to diffraction on the tetragonal nanodomains, which persists in the cubic matrix even up to 150 °C and was commented on in connection with Figure 7d [26,39,40]. For the prepared nano-BTO samples, the tetragonal imprint, although suggested by the small width of the 111 peak, is more difficult to detect due to its overlapping with other *hkl*-dependent broadening effects.

**Figure 10:**
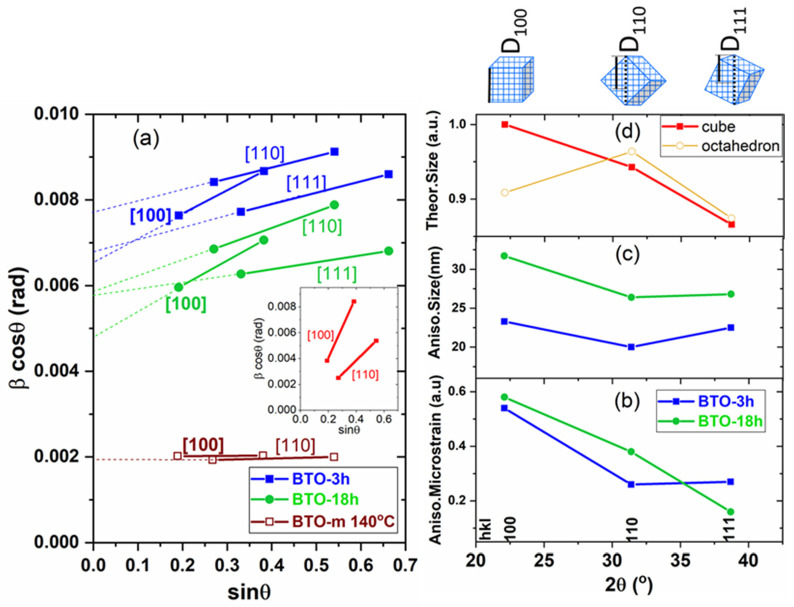
Williamson-Hall plots (**a**) and the results provided by these plots for the microstrain (**b**) and for the crystallite size (**c**); the variation of crystallite sizes as a function of the crystallographic direction are compared with the theoretical dependence on hkl of the sizes for cubic and for octahedral crystallites represented in (**d**). Inset of Figure (**a**): Williamson-Hall plots considering that the peak widths are determined only by tetragonal split amplitudes.

For more insights, the XRD data were processed using the Williamson-Hall method [44], in an attempt to unveil both size and strain anisotropy in the nano-BTO samples. Size anisotropy refers to the different linear dimension of the crystal when measured in different directions (anisotropic crystallite shape). Anisotropic microstrain refers to different values of the interplanar spacing dispersion along different directions in the crystal. Both size and microstrain contribute to XRD line broadening. The separation of the two effects is based on their differing dependence on the diffraction angle, with size broadening being proportional to 1/cos*θ*, whereas microstrain broadening varies as tan*θ*. This method allows the determination of size and strain using the so-called Williamson–Hall plot. Taking reflections of different orders on a given crystal plane, the plots should be straight lines of which the y-intercept is λ/D*_hkl_*, and the slope is the microstrain. Figure 10a shows the Williamson–Hall plots drawn for the first and second-order line pairs corresponding to the planes (100), (110) and (111), the only pairs visible in the scanned angular range 2*θ* = 20–90°. This is the same as saying parallel to the [100], [110] and [111] directions because, in the cubic system, the [*hkl*] directions are always perpendicular to the (*hkl*) planes.

One can observe that for both nano-BTO samples, the slopes of the plots corresponding to the [100] crystallographic direction are clearly larger than for the other two directions, indicating a larger microstrain in this direction. One should note that the possible tetragonal split can also play an important role in determining the slopes of the lines, leading to false values of microstrain. This is illustrated in the inset of Figure 10a. The plots in this inset were obtained considering that the integral breadths of the peaks are equal to the split amplitudes represented in Figure 8f. Although this assumption is exaggerated, the plots indicate that the results obtained using the Williamson–Hall method should be treated with caution if tetragonal splitting is possible. In addition to the nano-powders, we also applied this method to BTO-m at 140 °C, where it is in the cubic phase. In this case, the slopes were almost zero, indicating a much smaller microstrain compared to the nano-BTO samples. The y-intercept and the slope of the line drawn by two points were determined by “linear fit”, and the results are presented in Table 4 and are graphically depicted in Figure 10b,c. The variation of crystallite sizes as a function of the crystallographic direction is compared with the variation of the theoretical sizes (the average column lengths) for cubic and octahedral crystallites, represented in Figure 10d. These values were calculated as the reciprocal of the values listed in Table 3. One can see that the dependence of sizes on *hkl* roughly agrees with the theoretical one for cubic crystallites and does not correlate with the plot corresponding to octahedral crystallites. This could be a confirmation of the predominant cubic shape of the crystallites. It is worth notice that the cube side (D_100_) is larger than the average sizes obtained by Pawley fitting supposing isotropic crystallite sizes. This is because the microstrain is the greatest perpendicular to the cube faces. We also included these values in Table 1 and Figure 5.

The fact that microstrain is considerably larger parallel to the cube sides, <100>, than along the face diagonals, <110>, or space diagonals, <111>, means that the dispersion of the relative value of the lattice constant, <(Δ*a/a*)^2^> ^1/2^, is larger than the dispersion of <(Δ*d/d*)^2^>^1/2^ in other directions. This can be associated with the surface relaxation of a cubic crystal bounded by {100} faces. Surface relaxation refers to the modification of the interatomic distances perpendicular to the surface near the crystal surface. Heifets et al. [30] investigated the surface relaxation of cubic BaTiO_3_ using the shell model and found that Ti^4+^, Ba^2+^ and O^2−^ ions move differently from their crystal sites, leading to a dipole moment at the surface, perpendicular to the surface. Their results showed that the influence of the surface extends on 5–6 atomic layers below the crystal surface, which means 2–3 unit cells. The displacement of the atomic layers presumably leads to the lattice period’s variation perpendicular to the surface. In addition to the increase in microstrain, surface relaxation also produces an increase in the average lattice constant value. By combining theoretical modelling with experimental XRD data and supposing an exponential decay of the relaxation from the surface, Ishikawa and Uemori observed that in a BaTiO_3_ crystal with a cubic structure and a cubic shape bounded by {100} faces, the maximum increase in the lattice parameter at the surface was Δ*a* = 0.15 Å, which decreased to 1/e at 8 unit cells from the crystal surface [29]. The surface relaxation does not depend on crystallite size, but the smaller the crystallite size, the higher the effect on both the average lattice parameter and the lattice parameter dispersion, and thus upon the <100> microstrain. We obtained a bit larger lattice constant for BTO-3h, which has smaller crystallites, than for BTO-18h (Figure 5 and Figure 7a), a result favorable to the surface relaxation model. However, the microstrain values do not agree with the expectations assuming this structure model (Figure 5 and Figure 10b).

**Figure 11:**
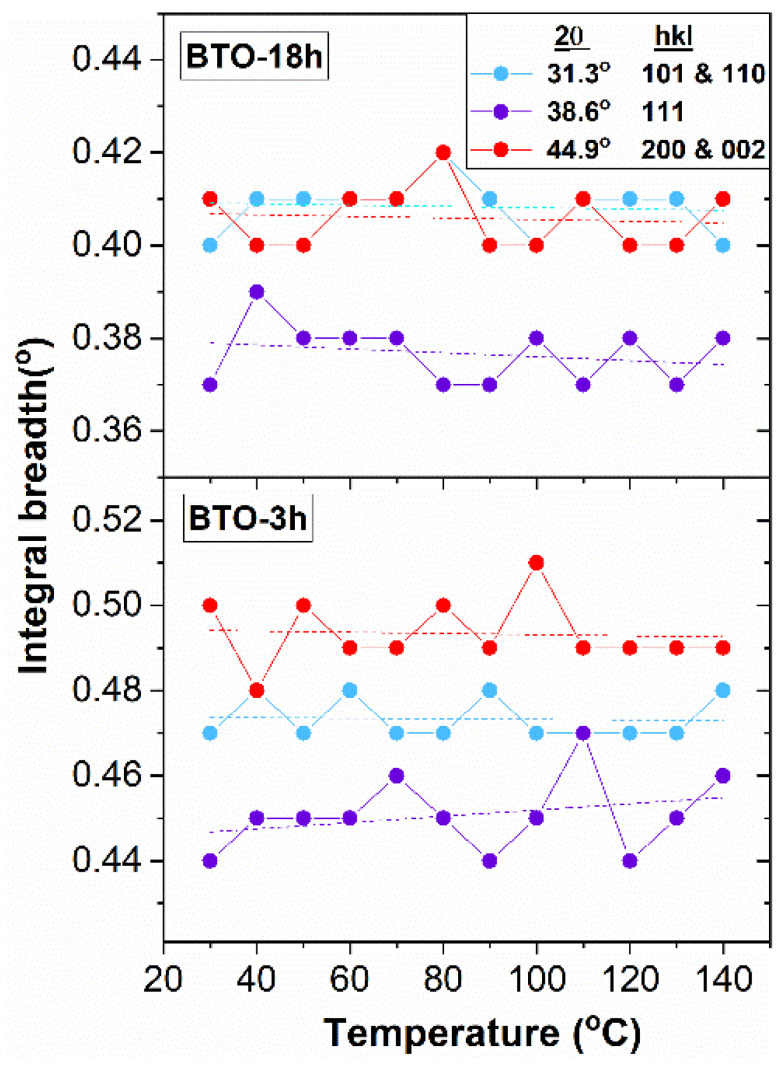
The XRD peak widths as a function of temperature for the nano-BTO samples. The large scattering of the experimental points is due to the small differences between the peak widths.

The widths of the three peaks scanned in the temperature-dependent measurements remain practically constant with temperature (Figure 11). This indicates, with high probability, that the anisotropy characteristics presented in Figure 10 are preserved in the whole temperature range investigated. If the large microstrain in the <100> directions is indeed a sign of surface relaxation, then it follows that the magnitude of surface relaxation is also preserved in the temperature range investigated.

### 3.3. Raman Analysis

**Figure 12:**
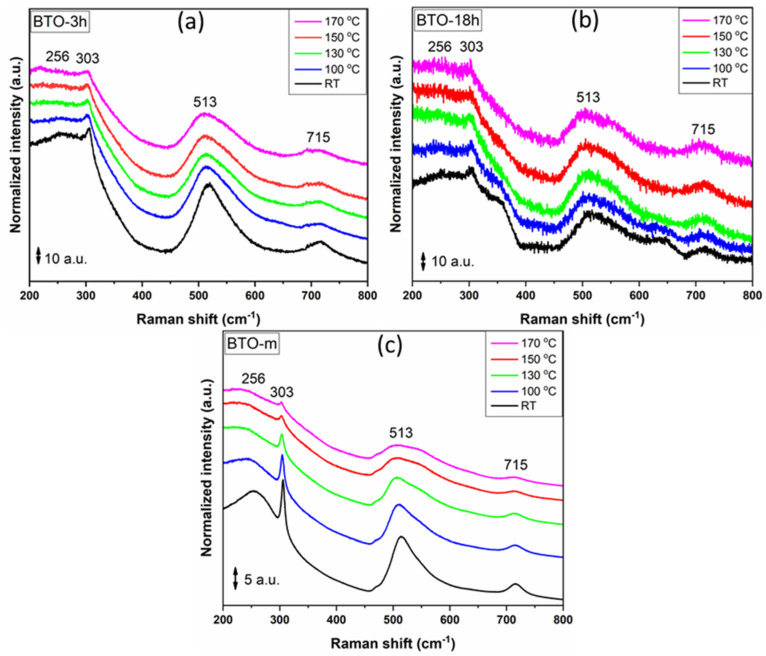
Raman spectra for the studied samples (**a**) BTO-3h, (**b**) BTO-18h and (**c**) BTO-m. The spectra were shifted vertically.

Raman spectroscopy was used to characterize the local structural distortions existing in the nano-BTO prepared (BTO-3h and BTO-18h) and BTO-m samples. The temperature-dependent Raman spectra of the studied BTO nanocrystals are depicted in Figure 12.

Due to the isotropic distribution of the electrostatic forces (Oh symmetry) around the Ti^4+^ ions, associated with four degenerate 3F_1u_ + F_2u_ phonons, in cubic BaTiO_3_ remains Raman-inactive [45]. However, when the cubic symmetry is distorted and the titanium centers suffer an off-center shift, the symmetry of the lattice will be lowered to tetragonal symmetry. Therefore, the tetragonal structure will lead to a splitting of the four degenerate phonons (3F_1u_ + F_2u_) into eight Raman-active phonons (longitudinal (LO) and transverse (TO)) responsible for the observed Raman scattering [46,47]. In this study, the assignment of the vibrational bands described below to specific vibrational modes of BTO were carried out on the basis of previous Raman investigations of single-crystal [46,48,49], bulk [47,50,51,52], thin-film [53] and nanocrystalline BTO [25,51].

In our case, at RT, the Raman spectra presented clear spectral lines located around 256, 303, 513 and 715 cm^−1^, indicating the presence of local tetragonal distortions in the corresponding nanocrystals. The 256 and 303 cm^−1^ spectral lines were due to the A_1_(TO_2_) and E(TO_2_) phonons propagating in non-centrosymmetric tetragonal BTO [52], whereas the 715 cm^−1^ line was associated with a tetragonal lattice and it was assigned to A_1_(LO_3_) phonons. These spectral lines decrease slightly in amplitude with the increase of the temperature above 130 °C, indicating a possible phase transition from a tetragonal to a cubic lattice (see Figure 12a,b) [26]. Moreover, it was already established [54] that a correlation between peak intensity and tetragonal features exists, emphasizing that a decrease in scattering intensity is related with the decline of the tetragonal phase and the formation of a cubic phase.

For BTO-m, the tetragonal phase lines showed a considerable decrease in intensity between 100 °C and 130 °C, continuing to decrease slowly up to the maximum temperature at which the measurements were carried out, but did not completely disappear (Figure 12c).

## 4. Discussion

The surface relaxation of the cubic lattice of nano-BTO involves modification of the lattice symmetry near the surface, with a larger mean period perpendicular to the surface than in a parallel direction. The gradient tetragonal-like structure is non-centrosymmetric but different from the bulk tetragonal one [30]. The tetragonal structure in our nano-BTO samples was clearly revealed by Raman analysis (Figure 12) and suggested by the narrow 111 XRD peaks (Figure 8 and Figure 11). The surface relaxation model is supported by the increased microstrain in the cube-side direction. Observing that the widths of XRD peaks (which are related, apart from the size of the crystallites, to the microstrain anisotropy and tetragonal deformation) did not change significantly with temperature, just as the Raman peaks corresponding to the tetragonal phase, one can conclude that the surface relaxation determines the tetragonal deformation.

In BTO-m, surface relaxation has a much lower weight due to the much larger size of the crystallites. XRD and Raman showed that, in this case, the tetragonal footprint decreases significantly with temperature. We propose that the strong tetragonal imprint visible at temperatures above the tetragonal-cubic transition temperature originates from the nanometer size domains with intermediate structure between the tetragonal and cubic BTO, which are paraelectric, occur around the phase transition temperature near the surface of the single-domain crystallites, and persist up to more than 140 °C [26,38,39,40].

**Figure 13:**
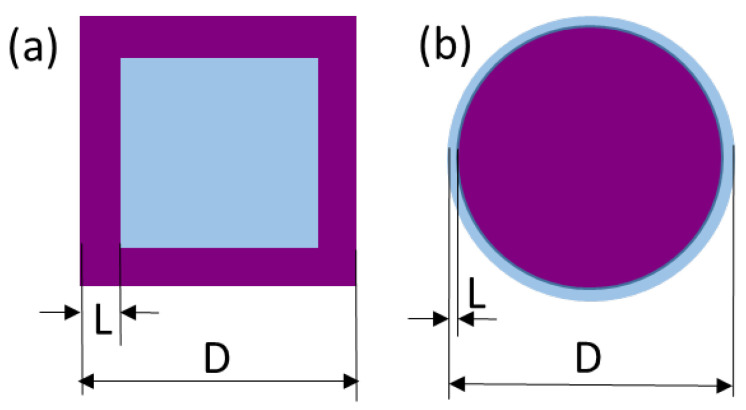
Sketches of the crystallites in the core-shell model; (**a**) nano-BTO, (**b**) BTO-m; blue—cubic phase, purple—tetragonal phase.

The experimental results obtained in this study can be interpreted within the core-shell model both for the nano-BTO samples and BTO-m. The core has a cubic structure in the first case and a tetragonal one in the second, and vice versa for the shell (Figure 13). Some quantitative estimations can be inferred, to be compared with the experimental results. For both cubic and spherical crystallite, the volume fraction of the core is given by the relation:

(1) $$core\%=100\;{\left(1-\frac{2L}{D}\right)}^{3},$$

where *D* is the linear size of the grain and *L* is the shell thickness. Conversely, knowing the weight of the core and the grain size, one can calculate the thickness of the shell.

If the shell is a layer with a modified structure due to the surface relaxation, then the shell thickness is approximately L = 3.5 nm (about 8 unit cells) for nano-BTO [29] and 5–15 nm for BTO-m [27,28]. Applying Equation (1), one can calculate the volume percentage of the majority core structure phase for the studied samples.

**Figure 14:**
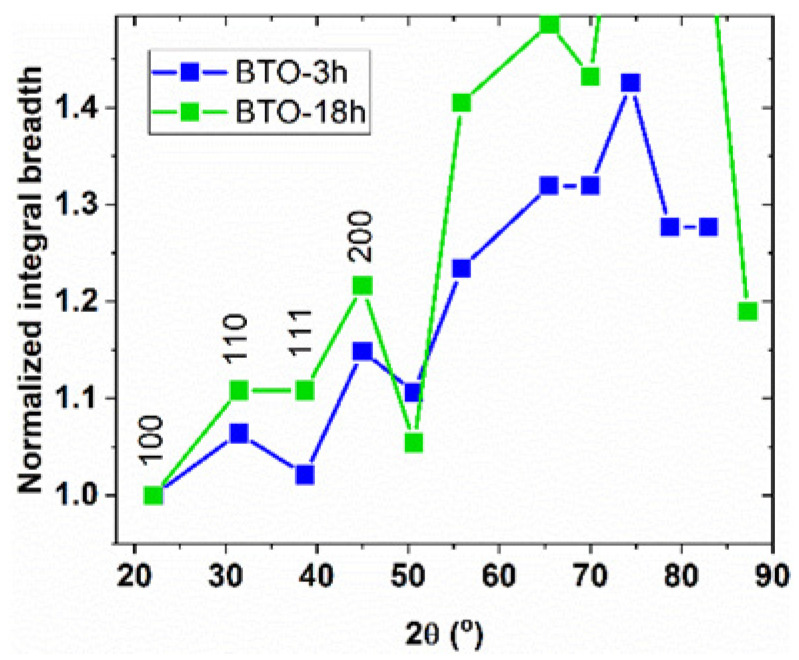
The peak widths of the prepared nano-BTO powders, normalized to the 100 peak width.

In the case of nano-BTO crystallites with D = 18 nm (sample BTO-3h) and D = 24 nm (sample BTO-18h), one obtains that the tetragonal phase (shell) volume percentage is 77% and 64%, respectively. Hence, the tetragonally deformed structure should be considerably abundant in these samples and more in BTO-3h than in BTO-18h. As we stated above, the tetragonal imprint can be assigned to the narrow 111 line, which is a single peak in the tetragonal phase, compared to neighboring peaks 110 and 200, split in the tetragonal phase. Figure 8a suggests that the tetragonal imprint is stronger for BTO-3h than for BTO-18h, but it is more correct to compare the relative line breadths of the two samples, as represented in Figure 14, in which the peak widths were normalized to the width of the 100 peak. These graphs clearly show that the tetragonal imprint is stronger for the smaller-sized BTO. The fact that the contribution of the tetragonally deformed surface structure increases with the size decrease is in agreement with the trend of increasing distortion in Ba-Ti distances and titanium off-centering with decreasing particle size, as established by Smith et al. [25].

Concerning the mesoscale BTO sample, BTO-m, with a majority tetragonal structure at room temperature, and a crystallite size of about 120 nm, Equation (1) shows that the tetragonal phase weight should be about 42–77 vol%. When assuming a two-phase structure (tetragonal + cubic), we estimated by Rietveld fitting, the tetragonal phase at room temperature to be about 78–79 vol% (wt% and vol% are equal in this case because the unit cells of two phases have the same composition and almost the same volume), which corresponds according to Equation (1) to a shell thickness of about 5 nm. On the other hand, assuming that the interpretation of the increase in crystallite size with temperature is correct (see comments for Figure 7d), the shell thickness is about 10 nm. Given that the fit quality in the case of BTO-m is quite poor due to the approximations made in both the Pawley and Rietveld fitting methods, the results matter more qualitatively than quantitatively (except for the lattice constants). Therefore, one can conclude that the core-shell model also matches well in this case.

In conclusion, the results of this study support the core-shell model of BTO, with a shell with a distorted structure due to surface relaxation.

## 5. Conclusions

A cube shape, one of the most desired morphologies of nanocrystals due to its ability to form close-packed structures in thin layers, was obtained using a solvothermal method at different reaction times. The synthesis of perfect cubic-shaped crystallites with a narrow size distribution and with no interstitial or adsorbed impurities is a challenging task. In this study, we succeeded in identifying some structural changes as a function of the reaction time. The sizes of the cuboid crystallites of the prepared nano-BTO samples, as revealed by XRD and TEM, ranged from about 18 to 24 nm, depending on the synthesis time. Assuming cubic perovskite structures for the prepared powders, Pawley fitting of the diffractograms yielded large lattice constants and large microstrain compared to a large crystallite size BTO, BTO-m. By taking into account the microstrain anisotropy, XRD was able to reveal the preponderant cube-shapes of the crystallites. The fingerprint of the tetragonal phase was revealed by observing the correlation between the peak widths and the tetragonal split amplitudes. This was most evident in the case of BTO-m above the transition temperature, in which the anisotropic size effect was absent. The large anisotropic microstrain shown by the nano-powders, which have a maximum value in the <100> directions, was considered evidence of the phenomenon of surface relaxation of cubic crystallites bounded by {100} faces. Raman spectroscopy showed characteristic tetragonal Raman lines for all samples. The intensities of these lines decrease only slightly with increasing temperature for the prepared nano-BTO samples. In the case of BTO-m of about 120 nm domain size, the tetragonal Raman lines severely decreased with increasing temperature above the tetragonal-cubic transition, but did not disappear even at 170 °C. This behavior is in agreement with the XRD results.

This study revealed non-obvious structural features of both nano-BTO and large crystallite BTO, using simple and easily accessible methods. We propose that applying the presented XRD methods, based on monitoring the peak width dependence on Miller indices, could be easily used to compare BTO nano-powders, for instance, to check their structural stability and obtain early diagnoses of changes in their structure.

## Figures and Tables

**Table 1:** The structure parameters obtained by means of Pawley whole pattern fitting, and the structure parameters of the samples obtained by XRD, assuming perfect cubic perovskite structures for the nano-BTO samples and single-phase tetragonal structure for BTO-m.

Sample	Crystal System	Crystallite (Domain) Size (nm) Min-Max ^1^	Microstrain (arb. units)	Lattice Constant (Å)
BTO-3h	Cubic	18.0 17.8–23.3	0.24	4.0264
BTO-18h	Cubic	23.9 22.9–31.7	0.29	4.0246
BTO-m	Tetrag.	118	0.16	a = 3.9964 c = 4.0320

^1^ The minimum values were obtained by fitting without microstrain; the maximum values were obtained by considering anisotropic microstrain (Section 3.2.2).

**Table 2:** The linear thermal expansion coefficients deduced from the variation of the lattice constant. We estimated the uncertainty of the thermal expansion coefficients based on the standard deviation of the slopes given by the linear fitting.

Sample	Crystallite Size (nm)	Linear Thermal Expansion Coefficient (10^−6^ K^−1^)	Thermal Expansion (%)	Increase in Crystallite Size (%)
BTO-3h	18.0	4.8 ± 5%	0.05	0.8
BTO-18h	23.9	3.4 ± 9%	0.04	1.7
BTO-m	118	1.0 ± 80% tetragonal phase 16 ± 10% cubic phase	0.01	22 (up to 110 °C)

**Table 3:** Values of the fraction (cube side)/(average column length perpendicular to the (*hkl*) planes) for a cube, and (octahedron edge-length)/(average column length perpendicular to the (*hkl*) planes) for an octahedron [42].

Reflection (Cubic Lattice)	Cubes (Bounded by {100} Faces)	Octahedrons (Bounded by {111} Faces)
100	1.0000	1.1006
110	1.0607	1.0376
111	1.1547	1.1438
210	1.0733	1.1075
211	1.1527	1.1061
221	1.1429	1.1185
310	1.0672	1.1138
311	1.1359	1.1211

**Table 4:** Results obtained using the Williamson–Hall method.

Sample Direction	Size (nm)	Microstrain (a.u.)
BTO-3h		
<100>	23.3	0.54
<110>	20.0	0.26
<111>	22.5	0.27
BTO-18h		
<100>	31.7	0.58
<110>	26.4	0.38
<111>	26.8	0.16

## Data Availability

Not applicable.
